# Granular cell tumors of the tongue: fibroma or schwannoma

**DOI:** 10.1186/s13005-017-0158-9

**Published:** 2018-01-03

**Authors:** Atsushi Musha, Masaru Ogawa, Satoshi Yokoo

**Affiliations:** Department of Oral and Maxillofacial Surgery, Plastic Surgery, Gunma University Graduate School of Medicine, Gunma, Japan

**Keywords:** Granular cell tumor, Oral cavity, Immunohistochemistry, Wallerian degeneration

## Abstract

**Background:**

Granular cell tumors are benign lesions that typically occur in the oral cavity, but can also be found in other sites. However, the characteristics of these tumors are unclear. Thus, the present study aimed to investigate the immunohistological characteristics of these tumors of the tongue.

**Methods:**

Seven patients were treated for granular cell tumors of the tongue at our institution during 2003–2017. Paraffin-embedded specimens were available for all cases; thus, retrospective immunohistochemical analyses were performed.

**Results:**

All cases exhibited cytoplasmic acidophilic granules in the muscle layer of the tumor. Both the normal nerve cells and tumor cells also stained positive for PGP9.5, NSE, calretinin, and GFAP. A nucleus of tumor cells was typically present in the margin. The PAS-positive granules were also positive for CD68 (a lysozyme glycoprotein marker). Various sizes of nerve fibers were observed in each tumor, and granular cells were observed in the nerve fibers of a representative case.

**Conclusions:**

Based on our immunohistological findings, granular cell tumors may be derived from Schwann cells, and the presence of CD68 indicates that Wallerian degeneration after nerve injury may be a contributor to tumor formation. Thus, a safe surgical margin is needed to detect the infiltrative growth of granular cell tumors.

## Background

Granular cell tumors (GCTs) are rare, benign tumors that were first reported as granular cell myoblastomas in 1926 [1]. The term GCT was first introduced in the 2005 version of the World Health Organization’s Classification of Tumors [2]. Contrary to the belief that GCTs have a myogenic origin, an immunohistochemical study [3] has revealed that GCTs are of neural origin, with diffuse expression of S-100 protein present in almost every case. However, there is no clear consensus regarding the mechanism of GCT development. GCTs display cytoplasmic acidophilic granule-like structures, and exhibit many polygonal neoplastic cells that multiply in an alveolar configuration. Because GCTs lack a capsule, they have poorly differentiated margins and frequently exhibit recurrence [4, 5]. Thus, curative treatment for GCTs requires a sufficient clear surgical margin. GCTs are frequently detected in soft tissues throughout the body, especially the skin and oral cavity [5], although the origin of this tumor remains unclear. Therefore, we aimed to evaluate the immunohistological characteristics of oral GCTs of the tongue.

## Methods

### Case selection

We retrospectively reviewed records from cases of oral GCTs of the tongue, treated in our hospital during a 15-year period (2003–2017), and identified 7 cases that had been treated by resection. The specimen submission forms were used to extract the data on patient’s age and sex, and the location of the lesion. Paraffin-embedded specimens were available for all cases, which allowed us to perform detailed histopathological and immunohistochemical analyses. All patients had provided informed consent for treatment, and this study’s retrospective design was approved by our Institutional Review Board (reference number: 150,033).

### Morphological assessment

Slides were stained with hematoxylin and eosin to evaluate the following morphological parameters: surgical margin status, presence of pseudocarcinomatous hyperplasia and cytoplasmic acidophilic granules. We also determined the presence of a capsule in these samples.

### Immunohistochemical analysis

We stained the slides with antibodies against the following proteins to determine the development mechanism and origin of the GCTs (Table 1):

**Table 1:** Characterization of the selected antibodies and their expression in granular cell tumors from various sites

Marker	Dilution	Supplier	Character
S-100 protein (rabbit polyclonal antibody)	1:300	DakoCytomation	E-F hand family (granule cells, glia cells, Schwann cells)
Vimentin (mouse monoclonal antibody)	1:300	DakoCytomation	Intermediary filament (granule cells)
PGP 9.5 (mouse monoclonal antibody)	1:300	DakoCytomation	Nerve cells (cell body, axon, granule cells)
NSE (mouse monoclonal antibody)	1:1000	DakoCytomation	Nerve cells (axis-cylinder process)
Calretinin (rabbit polyclonal antibody)	1:10	Spring Biosciences	E-F hand family (Schwann cells, central nerve neurons, mast cells)
GFAP (mouse monoclonal antibody)	1:1000	Novocastra Laboratories	Glia fiber-related acid protein, Schwann cells
CD68 (mouse monoclonal antibody))	1:300	Thermo Fisher Scientific	Highly glycosylated membrane proteins, glycoproteins
Ki-67 (mouse monoclonal antibody)	1:400	DakoCytomation	Proliferation marker

*Abbreviations*: *NSE* neuron-specific enolase, *GFAP* glial fibrillary acidic protein, *PGP* protein gene product

S-100, a protein from the E-F hand family and a marker widely used in immunostaining of GCTs [5]. The protein was extracted from the brain and is typically found in granule cells, glial cells and Schwann cells.

Vimentin, a protein from the intermediary filament family and associated with the cytoskeleton. It is typically found in granule cells, but has low specificity [5].

The PGP9.5 protein, is seen in nerve cells (cell body and axon) and neuroendocrine cells in the peripheral nervous system. This protein is extracted from the brain, and GCTs exhibit a broad range of staining intensities for PGP9.5 [5, 6].

The NSE protein is a marker of nerve cells (axis cylinder process) and neuroendocrine cells in the peripheral nervous system. This protein is highly specific for nerve cells (axis cylinder process) in all organs, and is produced in large quantities by neuroendocrine cell-derived tumors. Thus, NSE has been used for the diagnosis and monitoring of small-cell lung cancer, neuroblastoma, and neuroendocrine tumors [7].

The calretinin protein, an E-F hand family protein produced in the central nervous system (similar to S-100) [5]. Calretinin is typically found in Schwann cells, neurons of the central nervous system and mast cells.

The GFAP protein is associated with glial fiber-related acid protein and Schwann cells, and is an intermediate filament protein, specific to astroglial cells [8]. The expression of GFAP increases in cases of cerebral damage, dementia, prion disease, and neurologic diseases such as multiple sclerosis.

The CD68 protein is associated with highly glycosylated membrane proteins, glycoproteins, and mucin-like membrane proteins of lysozymes [9]. This protein is typically found in macrophages, fibroblasts, and Schwann cells.

The Ki-67 protein, a cell proliferation marker used to examine the proliferation status of tumor cells [10].

## Results

**Fig. 1:**
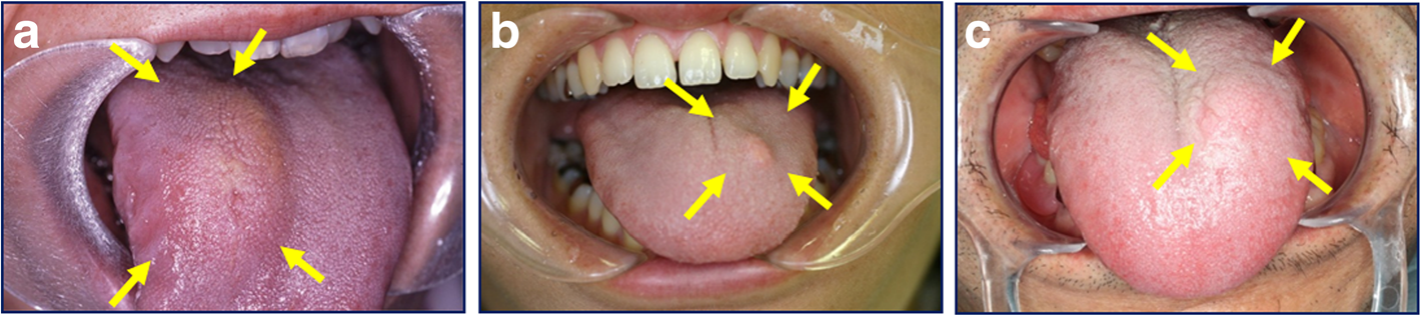
Granular cell tumors of the tongue (arrows). **a** Case 2, **b** Case 5, and **c** Case 6

All 7 GCTs occurred in the tongues of middle-aged or elderly patients, all of whom had presented with a hard lump in their tongue (Table 2, Fig. 1). The tumor dimensions ranged from 5 to 29 mm. In all cases, the clinical diagnosis was fibroma. No cases of recurrence were observed over a maximum follow-up period of 15 years.

**Table 2:** Clinical characteristics

Case	Age (years)	Sex	Major complaint	Location	Size (mm)	Mucosal color	Lingual trauma from occlusion	Follow-up (months)
1	45	F	Hard lump	Tongue	8 × 8	Normal	+	202
2	43	F	Hard lump	Tongue	20 × 29	Normal	+	177
3	53	F	Hard lump	Tongue	7 × 7	Normal	–	162
4	39	M	Hard lump	Tongue	5 × 5	Normal	–	147
5	35	F	Hard lump	Tongue	7 × 7	Normal	–	108
6	62	M	Hard lump	Tongue	16 × 17	Normal	+	65
7	70	F	Hard lump	Tongue	13 × 18	Normal	–	40

*Abbreviations*: *F* female, *M* male

All cases exhibited cytoplasmic acidophilic granules in the tumor muscle layer. A nucleus of tumor cells was typically present in the marginal regions. In none of the cases the lesion was covered by a capsule and only 3 cases exhibited pseudocarcinomatous hyperplasia. All surgical margins were >10 mm.

**Fig. 2:**
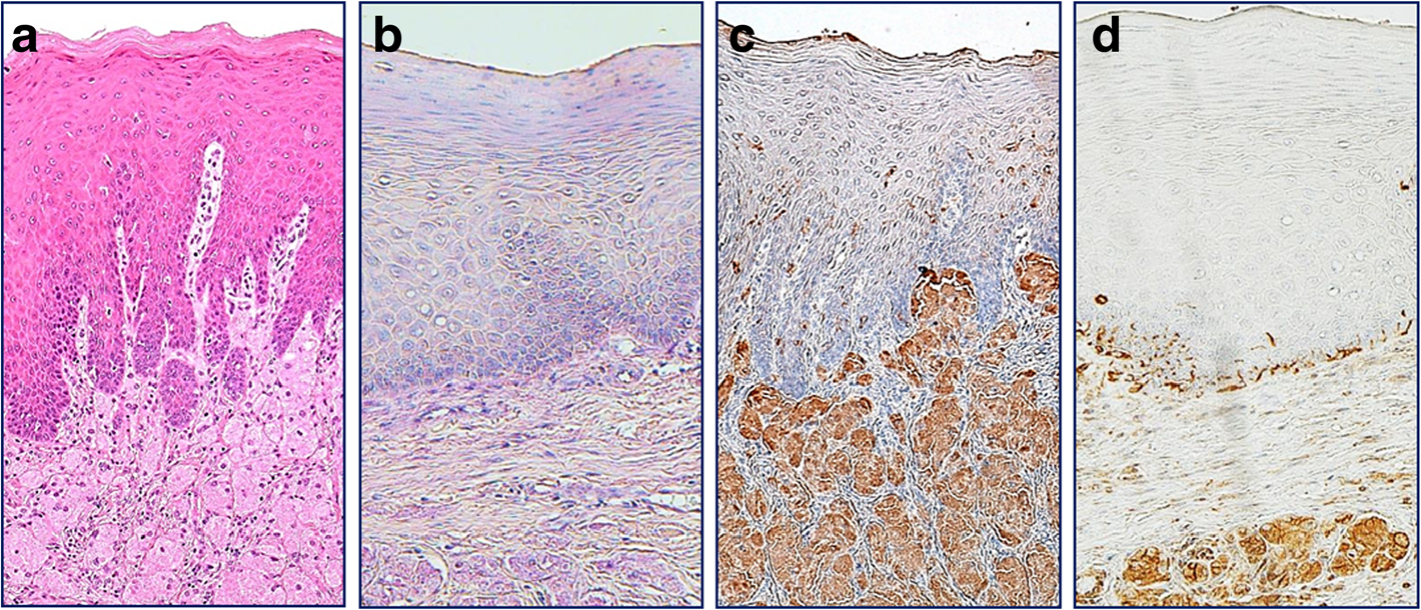
**a** Pseudoepitheliomatous hyperplasia associated with granular cell tumors (hematoxylin and eosin staining). All cases stained positive for **b** periodic acid-Schiff, **c** S-100 protein, and **d** vimentin (magnification, 100×)

**Fig. 3:**
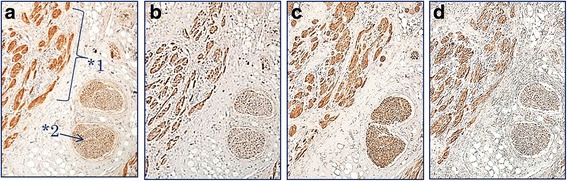
Immunohistochemistry revealed that the tumor cells (*1) and normal nerve cells (*2) expressed **a** protein gene product 9.5, **b** neuron-specific enolase, **c** calretinin, and **d** glial fibrillary acidic protein (magnification, 40×)

**Fig. 4:**
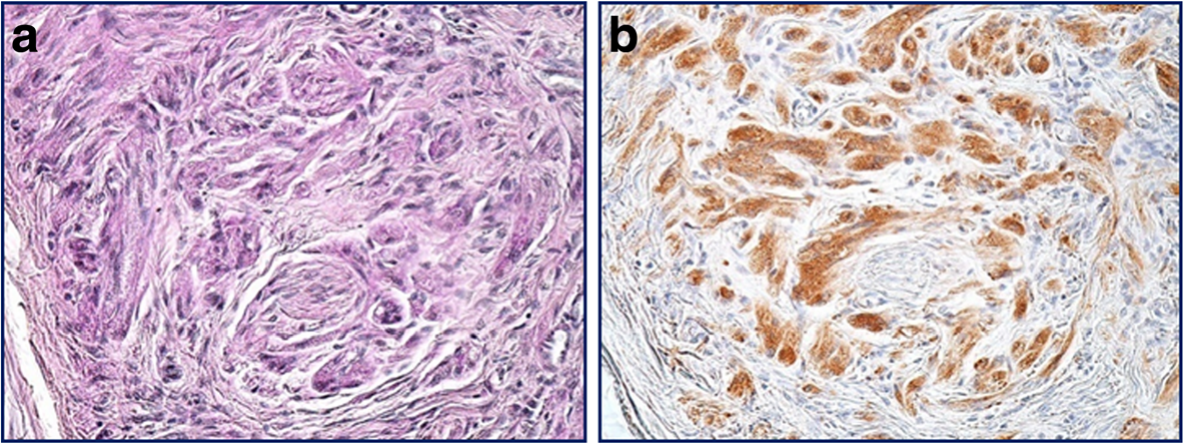
Granules stained positive for **a** periodic acid-Schiff and **b** the lysozyme glycoprotein marker, CD68 (magnification, 200×)

**Fig. 5:**
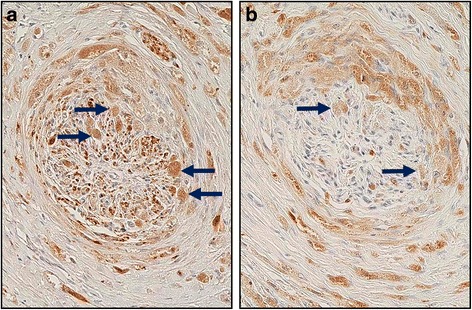
Granular cells (arrows) were observed in a nerve fiber from a representative case that was stained for **a** protein gene product 9.5 and **b** CD68 (magnification, 200×)

Immunohistochemical findings are summarized in Table 3. All cases stained positively for PAS, S-100 protein, vimentin, PGP 9.5, NSE, calretinin, GFAP, and CD68 (Figs. 2 and 3). Co-expression of PGP 9.5, NSE, calretinin, and GFAP in the tumor cells and normal nerve cells was observed in all cases (Fig. 3). The PAS-positive granules were also positive for the lysozyme glycoprotein marker, CD68 (Fig. 4). Nerve fibers of various sizes were observed in each tumor and granular cells were observed in the nerve fibers from a representative case (Case 2; Fig. 5). The tumors exhibited sporadic staining for Ki-67, with a mean Ki-67 index of 1.89% (low cell proliferation index; Table 3).

**Table 3:** Histopathological and immunohistochemical characteristics in 7 cases of GCT of the tongue

Case	Capsule	PCH	S-100 protein	Vimentin	PGP 9.5	NSE	Calretinin	GFAP	CD68	PAS	Ki-67 index (%)^a^
1	–	–	+	+	+	+	+	+	+	+	2.57
2	–	+	+	+	+	+	+	+	+	+	3.22
3	–	+	+	+	+	+	+	+	+	+	1.21
4	–	–	+	+	+	+	+	+	+	+	0.42
5	–	–	+	+	+	+	+	+	+	+	1.83
6	–	+	+	+	+	+	+	+	+	+	1.63
7	–	–	+	+	+	+	+	+	+	+	2.42

*Abbreviations GCT* granular cell tumors, *GFAP* glial fibrillary acidic protein, *NSE* neuron-specific enolase, *PAS* periodic acid-Schiff, *PCH* pseudocarcinomatous hyperplasia, *PGP* protein gene product

^a^The Ki-67 index was calculated as the percentage of positive cells in a minimum sample of 1000 cells. Mean Ki-67 index, 1.89%

## Discussion

GCTs are relatively rare benign tumors that can occur throughout the body. The tongue is involved in ≥60% of oral GCTs, although these tumors can also be found in the head and neck region, buccal mucosa, hard palate, lips and gingiva [3]. A previous study [11] indicated that women are more likely to develop GCTs than men and we observed similar distribution. It is difficult to confirm a clinical diagnosis of GCT, since these tumors do not have clear clinical characteristics. The first treatment of choice is surgical resection. Since the tumor does not have a capsule and presents with undefined borders, careful resection with clear margins is essential to avoid recurrence [11], an event observed in approximately 20% of cases because of the presence of a resected stump [12]. Although malignant changes (histologic evidence of vesicular nucleus with a prominent nucleolus, high mitotic activity, high nucleus-to-cytoplasm ratio and pleomorphism) are relatively rare, and seen in 1–2% of cases [13, 14], prevention of a malignant transformation may also aid in preventing recurrence. Thus, a broad surgical margin is needed to prevent recurrence. In the present study, the tumors had relatively small volumes, and a 10-mm margin, based on palpation of the tumor mass, was considered sufficient. It is unclear whether a 10-mm margin can be used for all GCTs, although a 10-mm margin from the palpated mass may be appropriate in cases of GCT with small sizes.

Several theories have been proposed regarding the origin of GCTs. In 1926 Abrikosoff described this tumor as a “granular cell myoblastoma”, because of the presence of striated muscle blast cells. However, different reports [15–18] have suggested different origins, such as myogenic, neurogenic, histiocytic and fibroblastic. Nevertheless, based on the presence of the highly specific S-100 protein, it is highly likely that Schwann cells are involved, which was first described in 1982, alongside glial cells [11], resulting in a name change for these tumors to GCT in the 2005 version of the World Health Organization’s Classification of Tumors [2].

In the present study, all cases exhibited PAS-positive granules in the tumor cells alongside positive staining for S-100 protein and vimentin (Fig. 2). Furthermore, the tumor cells and normal nerve cells exhibited co-expression of nerve markers PGP 9.5, NSE, calretinin, and GFAP (Fig. 3), suggesting that the nervous system plays a role in the development of this tumor. Besides, varying thicknesses of funiculi were present in all of the tumors and Schwann cells exhibited findings that were comparable to those of granule cells (Fig. 5). These findings suggest that GCTs may originate from Schwann cells.

**Fig. 6:**
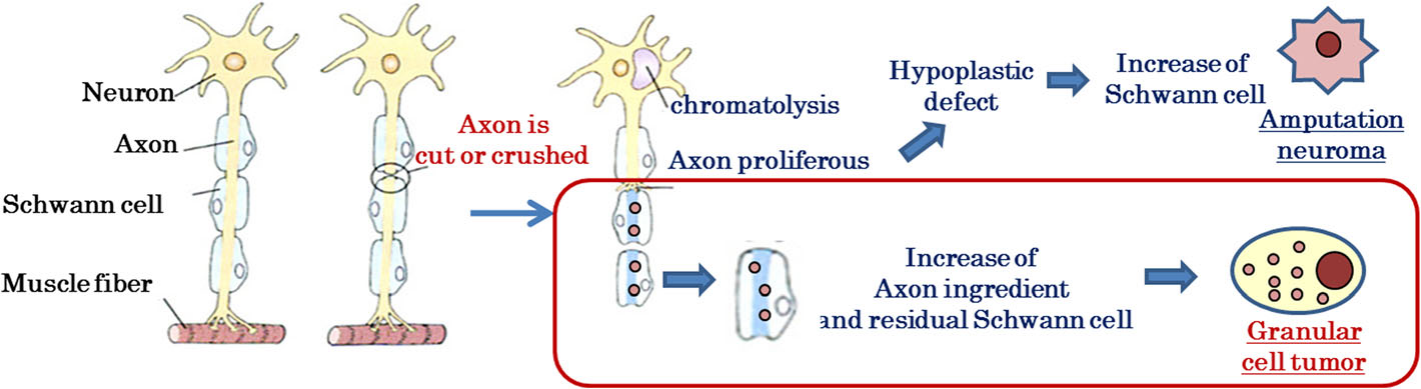
Regeneration of damaged axons may cause Wallerian degeneration. This process is induced after a nerve fiber is mechanically cut or crushed and results in distal degeneration of the axon after it is separated from the neuronal cell body. The axon fragment and residual Schwann cells may participate in the development of a granular cell tumor. Therefore, Wallerian degeneration may play an important role in the development of granular cell tumors

The cytoplasmic granular structures of the tumor cells are lysozymes, as they show positive staining for CD68 (Fig. 4), and are reported to contain glycogen, a myelin-like structure, and a phospholipid membrane [19]. As such, neurodegenerative injury has been suggested to be involved in the development and reproduction of GCT cells [20]. In our study, lingual trauma from occlusion was observed in 3 cases, leading to a conclusion that the development of GCT may involve Wallerian degeneration after axonal injury, which generates an axon fragment (i.e., glycogen and the myelin-like structure) with Schwann cells. This fragment may lead to a malignant transformation that can result in cancerous growth and the development of GCT (Fig. 6). An amputation neuroma may also be another cause. Though there are few reports of GCT occurring in such a situation, it is extremely unlikely that both will occur simultaneously. Hence, GCTs can arise in all soft tissues that might experience mechanical stimulation. It should be kept in mind that various other factors, besides Wallerian degeneration, may influence the developmental process in locations not exposed to mechanical injury (e.g., the lungs) [12].

All 7 cases exhibited a low Ki-67 index, which explained the favorable prognoses in our patients, with no signs of recurrence seen after a follow-up period of 15 years. Previous studies had revealed that a Ki-67 index of >10% was associated with local GCT recurrence [10].

## Conclusions

Our immunohistological findings suggest that GCTs are derived from Schwann cells. Furthermore, the CD68-positive findings indicate that Wallerian degeneration may also contribute to nerve injury. A safe surgical margin is needed to identify infiltrative growth of GCTs.

## Abbreviations

GCT: Granular cell tumors
GFAP: Glial fibrillary acidic protein
NSE: Nerves-specific enolase
PAS: Periodic acid Schiff
PGP: Protein gene product
